# Evidence of Cardiac Involvement in a Patient With Necrotizing Autoimmune Myopathy (NAM)

**DOI:** 10.7759/cureus.44106

**Published:** 2023-08-25

**Authors:** Omair A Khan, Rita M Wilches, Joseph N Mehrabi, Kurenai Tanji, Sarita Konka

**Affiliations:** 1 Internal Medicine, Maimonides Medical Center, New York, USA; 2 Resident Physician, Maimonides Medical Center, New York, USA; 3 Neuropathology, Columbia University, New York, USA; 4 Rheumatology, Maimonides Medical Center, New York, USA

**Keywords:** elevated troponin, bilateral limb weakness, inflammatory myopathy, immune-mediated necrotizing myopathy, non-st segment elevation myocardial infarction (nstemi)

## Abstract

Necrotizing autoimmune myopathy (NAM) is a rare inflammatory myopathy primarily affecting skeletal muscles. Cardiac involvement has been reported in immune-mediated necrotizing myopathy (IMNM), but its extent remains poorly understood. We present a unique case of a 68-year-old male with anti-signal recognition particle (SRP) antibody-positive NAM initially presenting with elevated troponin levels. Our case demonstrates cardiac involvement as the presenting feature of NAM, which is a unique feature of inflammatory myopathy.

## Introduction

Necrotizing autoimmune myopathy (NAM) is a rare yet severe condition characterized by muscle weakness and elevated creatine phosphokinase (CPK) levels. Diagnosis is confirmed through muscle biopsy and autoimmune workup, with specific autoantibodies such as anti-signal recognition particle (SRP) and anti-3-hydroxy-3-methylglutaryl-coenzyme A reductase (HMGCR) often present. Although cardiac involvement has been reported in immune-mediated necrotizing myopathy (IMNM), the extent of such involvement remains poorly documented [1,2]. Early recognition of NAM is crucial for timely treatment and improved treatment response outcomes.

## Case presentation

A 68-year-old male was referred to the emergency department by his primary care physician for bilateral lower extremity swelling, exertional chest pain, and elevated liver function tests (LFTs). The patient reported experiencing intermittent chest pain and shortness of breath for the last few months. The electrocardiogram (EKG) was normal, but a troponin level of 2 ng/mL was found on admission. Troponin levels were trended, but they remained the same (1.8, 2, 1.9). An echocardiogram revealed a left ventricular ejection fraction (LVEF) of 45-50% and hypomotility in the basal segments. A coronary angiogram performed at that time showed no significant coronary artery disease. The cardiology service diagnosed the patient with non-ST-elevation myocardial infarction (NSTEMI) and recommended further medical management. The patient had mildly elevated LFTs; hence, his statin medication was discontinued upon discharge, suspecting statin-induced myopathy.

One month later, the patient was readmitted to the hospital for progressive muscle weakness, both in the upper and lower extremities. He reported that symptoms began two months prior when he experienced leg weakness while walking and standing up from a seated position. A few weeks after the first hospital admission, the patient developed marked difficulty raising his arms, climbing stairs, and experienced difficulty in swallowing. One week before the second admission, he became unable to walk and was bedridden. The patient did not report proximal muscle weakness during the first admission.

**Figure 1:**
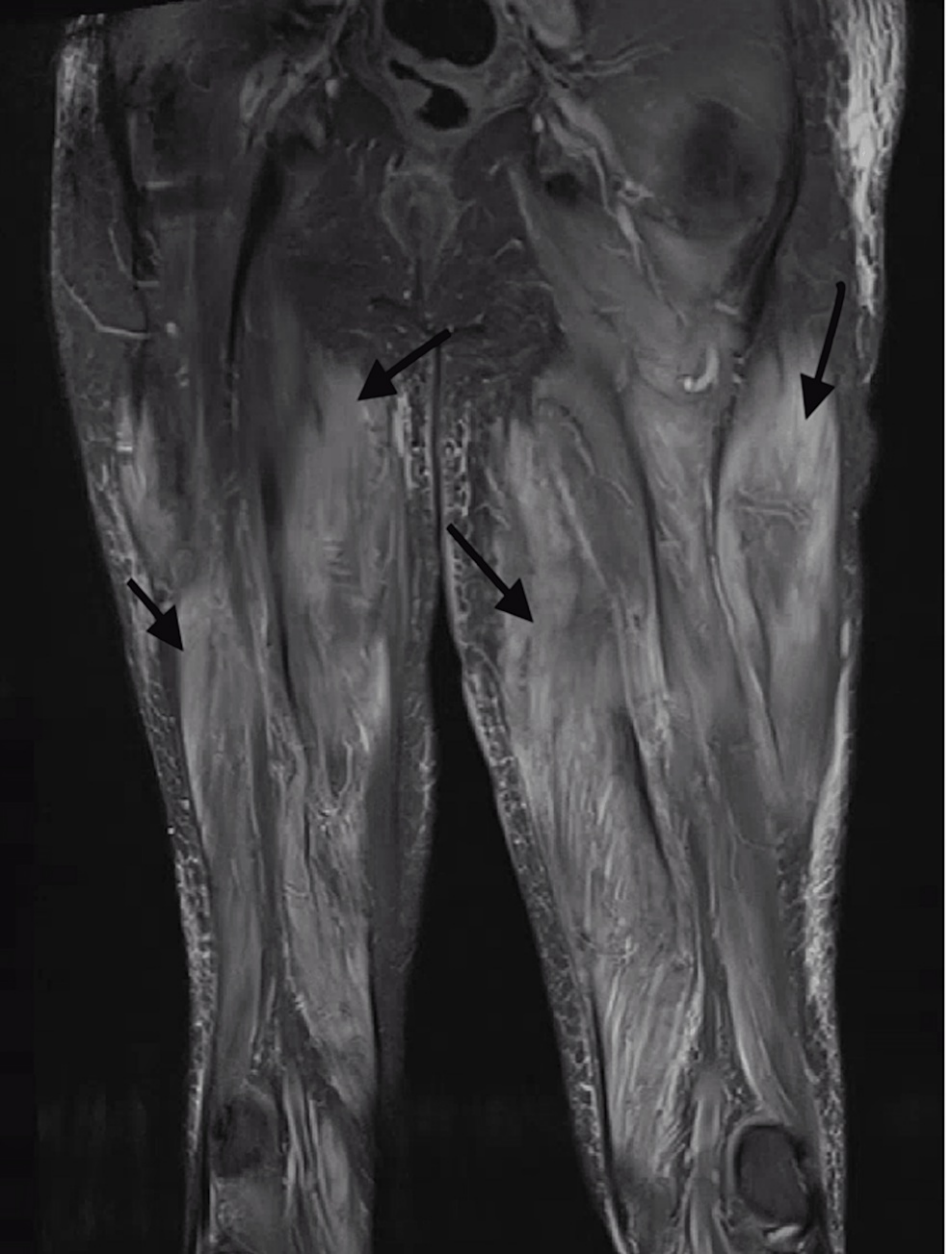
(Coronal STIR series): Extensive bilateral myositis (black arrows) more prominent in the anterior compartments STIR: short tau inversion recovery

**Figure 2:**
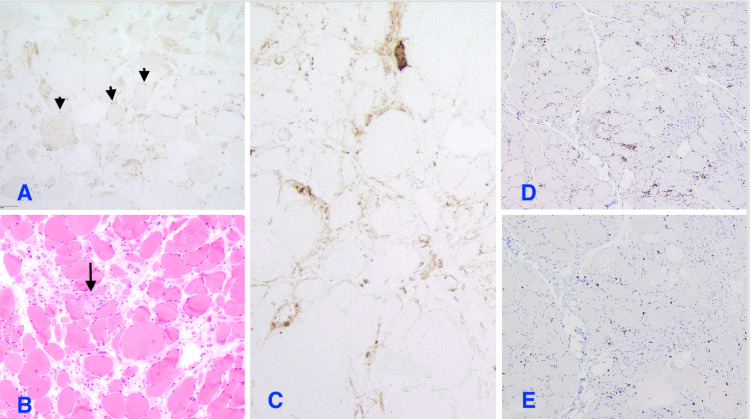
A: immunostaining for HLA-A, B, and C (HLA class 1) shows extremely faint sarcolemmmal immunoreactions in rare fibers [arrow heads] (original magnification x200); B: cryosection stained with Hematoxylin-Eosin shows marked variation in fiber size along with necrotic fibers [arrow]. Severe endomysial fibrosis is noted; C: immunostaining for MAC (membrane attack complex, C5b-9) shows sarcolemmal immune expressions in a subset of fibers with various staining intensity (original magnification x200); D: immunohistochemical staining for CD68 (paraffin section, original magnification x100), an inflammatory reaction was largely associated with myonecrosis. Infiltrates were mainly composed of CD68(+) histiocytes and a smaller number of CD3(+) T-cells. B-cells were inconspicuous; E: immunohistochemical staining for CD3 (paraffin section, original magnification x100)

Physical examination revealed significant proximal muscle weakness (3/5) in both upper and lower extremities. Laboratory tests showed CPK of 8200 IU/L (59-367), aldolase of 78 U/L (3.3-10.3), lactate dehydrogenase (LDH) of 1163 IU/L (108-199), alanine transaminase (ALT) of 157 IU/L (6-47), and aspartate aminotransferase (AST) of 257 IU/L (10-33). Erythrocyte sedimentation rate (ESR) and C-reactive protein (CRP) were normal. The patient had a troponin level of 1.83 ng/mL, which subsequently down-trended to 1.5. A repeat echocardiogram was done, which showed an ejection fraction (EF) of 60-65%, which had improved as compared to his last echo one month ago. There was suspicion of myocarditis, but it was later ruled out based on a normal cardiac MRI, which also failed to show any underlying infarction. The endoscopy performed to investigate dysphagia was unremarkable. An MRI of the thigh demonstrated extensive bilateral myositis (Figure 1), prompting a muscle biopsy that revealed an active chronic myopathy with necrotizing features (Figure 2). The patient's autoimmune workup returned a positive anti-SRP antibody, which is consistent with NAM. The patient was discharged on high-dose steroids. On four and eight weeks of follow-up, the patient continued to have weakness in his extremities; hence, mycophenolate mofetil was started, which had to be discontinued as he developed a drug-induced rash in the groin. He was subsequently started on methotrexate and intravenous immunoglobulin (IVIG), to which he responded well with significant improvement in muscle weakness. He continues to be monitored regularly for any signs of disease progression. His troponin level was repeated once after discharge, and it was positive at 2 ng/mL. 

## Discussion

NAM is a form of autoimmune myopathy characterized by pronounced proximal muscle weakness, muscle fiber necrosis with limited inflammatory cell infiltration on muscle biopsy, and rare involvement of areas beyond the muscles [1]. NAM has been associated with two main autoantibodies, namely anti-HMGCR antibody and anti-SRP antibody, and is classified into three distinct subtypes, which are anti-SRP, anti-HMGCR, and anti-HMGCR/SRP negative [1]. While NAM can be a sole entity, it may also be associated with connective tissue disorders and paraneoplastic complications. Alternatively, it can be idiopathic, manifesting without myositis-specific or myositis-associated antibodies or cancer [2].

The prevalence of anti-HMGCR antibodies in NAM has been reported to range from 22 to 61%. Typically, anti-HMGCR is associated with statin use, with up to 60% of statin-exposed patients with NAM found to have these antibodies [2]. However, it is crucial to distinguish anti-HMGCR myopathy from the more common toxic myopathy associated with statin use, as the latter improves with the discontinuation of the statin. Anti-HMGCR antibodies have also been reported in children and adults without exposure to statin therapy but with underlying malignancies [2]. In contrast, anti-SRP antibodies have been reported in up to 16% of patients with NAM who were found to be unresponsive to treatment [2]. Additionally, anti-SRP myopathy is more frequent in women and has been linked to abnormal EKG and echocardiogram results [1,3]. Moreover, compared to anti-HMGCR myopathy, anti-SRP myopathy can lead to severe weakness and has a higher risk of pulmonary involvement or dysphagia associated with it [2,4]. Although there is limited data on the incidence of cardiac involvement in NAM, inflammatory cardiomyopathy, or myocarditis, has been well documented in other autoimmune myopathies [1,5].

The prevalence of cardiac involvement in anti-SRP antibody-positive myopathy has been debated in previous reports and continues to be a controversial topic. In 1990, Targoff et al. reported that four out of 13 patients with anti-SRP antibody-positive myositis experienced cardiac involvement, including arrhythmias, heart failure, and cardiac fibrosis [6]. Additionally, Love et al. found that all seven cases in their study with anti-SRP antibodies presented with palpitations [3]. In contrast, Hengstman et al. reported in 2006 that less than 20% of patients with anti-SRP antibody-positive myopathy exhibited heart failure [7]. Similarly, Suzuki et al. identified cardiac involvement in only two of 100 patients with anti-SRP antibody-positive NAM [8]. A recent study in 2023 found that the prevalence of cardiac involvement in anti-SRP antibody myopathy was 50% (16/32) and included seven patients with acute coronary syndrome [9]. Takeguchi-Kikuchi et al. demonstrated an association between anti-SRP antibody-positive NAM and cardiomyopathy, as proven by positive myocardial biopsy, cardiac MRI, and fluorodeoxyglucose-positron emission tomography [10]. In recent years, Xeu Ma et al. reported a case of cardiomyopathy three years after the diagnosis of NAM; the patient ultimately ended up getting a heart transplant [11]. However, as per our knowledge, there is only one reported case of anti-SRP-positive NAM in which the patient initially presented to the hospital with cardiac manifestations. In that case, the patient was admitted with heart failure and was diagnosed with NAM during the same admission [12].

Diagnosis requires tissue confirmation by muscle biopsy, which reveals scattered necrotic muscle fibers undergoing myophagocytosis and inflammation localized to necrotic fibers [1,2]. In addition, diffuse expression of membrane attack complex (MAC) and major histocompatibility complex class I (MHC-1) on the sarcolemma of non-necrotic muscle fibers and deposition of MAC on small blood vessels can be observed [1,2]. The biopsy should be assessed for inflammation or other features to rule out alternative diagnoses. Then, serological testing for anti-HMGCR and anti-SRP antibodies is essential for a definitive diagnosis and guiding treatment [1,2].

Corticosteroids are a first-line treatment, but some patients may require the addition of a second-line agent due to either an incomplete response to steroids or intolerable side effects [1,2]. In cases of anti-HMGCR myopathy, IVIG monotherapy may be used as a first-line treatment [13]. Methotrexate, azathioprine, and mycophenolate mofetil are preferred second-line agents. Rituximab and IVIG are usually reserved for severe cases. In 2016, the European Neuromuscular Centre (ENMC) recommended that rituximab be added for all anti-SRP myositis patients within six months if other measures are ineffective and used instead of or in addition to methotrexate as second-line therapy [2].

We acknowledge the limitation of our case in establishing a causal association between NAM and the NSTEMI episode due to the low probability of performing a more invasive diagnostic tool, such as an endomyocardial biopsy. However, exertional chest pain with intermittent shortness of breath, persistent troponin elevation, and improvement of LVEF found on repeat echocardiogram support cardiac involvement as the presenting feature of NAM.

## Conclusions

Our case report presents a unique instance of necrotizing autoimmune myopathy (NAM) with an initial presentation of cardiac involvement with concern for non-ST-elevation myocardial infarction (NSTEMI), highlighting the importance of myocardial injury as a potential presenting feature of NAM. As NAM predominantly affects skeletal muscles, there is limited data regarding the incidence of cardiac involvement in this condition. Nevertheless, inflammatory cardiomyopathies have been documented as a major cause of mortality in other autoimmune myopathies. Prospective studies are needed to monitor asymptomatic cardiac involvement and assess the potential progression to dilated cardiomyopathy and decompensated heart failure. For patients with NAM, early cardiac screening, management, and follow-up are associated with better outcomes.
